# Concordance of a Structured Orthopantomogram Reporting Tool Between Dental Practitioners, and Compared to Routine Reports Within a Regional and Remote Service

**DOI:** 10.1002/cre2.70366

**Published:** 2026-07-16

**Authors:** Stevie Dilley, Lynlee Tatnell, Paul Monsour, Jacob O'Gorman, Megan J. Hobbs

**Affiliations:** ^1^ Royal Flying Doctor Service (Queensland Section) Brisbane Queensland Australia; ^2^ School of Dentistry University of Queensland Brisbane Queensland Australia; ^3^ School of Rural Medicine University of New England Armidale New South Wales Australia; ^4^ Clinical Research Unit for Anxiety and Depression University of New South Wales Sydney New South Wales Australia

**Keywords:** dental, health services accessibility, oral health, radiography, rural health services

## Abstract

**Objectives:**

Variability in interpreting orthopantomograms can have greater impact on regional and remote patients who experience poorer oral health, have reduced service access and incur greater costs, if referred to out‐of‐community services. Structured orthopantomogram reporting and specialist advice pathways may support more consistent image interpretation and clinical decision‐making. This study evaluated the agreement of a structured orthopantomogram reporting tool between dentists and compared the information recorded using the tool with routine clinical reports.

**Study Design:**

Pilot inter‐rater concordance study.

**Material and Methods:**

The inter‐rater and inter‐method agreement relating to image quality, radiographic features, and the need for specialist review were assessed across 50 orthopantomogra.

**Results:**

The structured tool was completed, on average, in 10.25 min (SD = 8.62), and yielded almost perfect agreement between the two dentists (*κ*(95%CI) = 0.89 [0.86–0.92]). Agreement across radiographic features ranged from substantial to perfect agreement, with lower agreement observed for whether a specialist referral was needed. Inter‐method agreement between the structured tool and routine reports was fair (*κ*(95%CI) = 0.33 [0.29–0.37]), likely reflecting the greater level of detail prompted by the structured tool rather than differences in the quality of routine reporting practices. Half of the orthopantomograms were reviewed by a specialist, who determined only 6% warranted a referral.

**Conclusions:**

Structured reporting supports more consistent interpretation of orthopantomograms. A formalised specialist advice pathway may reduce unnecessary referrals to out‐of‐community services, and support dentists' confidence and consistency when completing orthopantomogram reports.

## 1 Introduction

Oral health is a significant public health challenge globally and in Australia (Bernabe et al. 2020; James et al. 2018; Australian Institute of Health and Welfare 2023a) with caries and periodontal disease among the leading causes of disability (Australian Institute of Health and Welfare 2023a). Australians living in regional and remote areas experience a disproportionate burden with higher rates of dental disease and poorer overall oral health than those living in major cities (Australian Government Department of Health 2012; Australian Institute of Health and Welfare 2007; Crocombe et al. 2013). This inequity reflects persistent geographic workforce shortages with the number of full‐time equivalent dental practitioners per 100,000 residents reducing with increasing remoteness: a trend that has not changed in more than a decade (Australian Institute of Health and Welfare 2023b). This structural barrier reduces opportunities for preventative care, increases reliance on acute care, (Crocombe et al. 2013) and means that families often have to travel long distances, take time away from work, and incur additional costs when specialist referral is required out‐of‐community (Curtis et al. 2007). Perceived barriers also include direct service costs, private dental insurance, fear of dentists, and waitlist times (Mariño et al. 2014, 2021; Ghanbarzadegan et al. 2025).

To address these inequities, the RFDS (Queensland Section) Mobile Dental Unit provides what is often the only regular source of local dental care for many outer regional and remote Queensland communities. Operating for over a decade, it delivers comprehensive acute, preventative and restorative care along with community education to otherwise underserved communities. For instance, the Mobile Dental Unit, which is an 18‐wheel semi‐trailer that houses two modern dental surgeries, a sterilisation room and reception area, in 2022/2023, held more than 1780 initial and follow‐up consultations in 15 regional and remote communities (Royal Flying Doctor Service Queensland Section 2023). The RFDS (Queensland Section) is committed to returning to these communities annually to ensure ongoing access to dental care.

As part of Mobile Dental Unit consultations, dentists often take orthopantomograms (i.e., full mouth radiographs). These images have the benefit of providing a single full mouth radiograph demonstrating the dentition, upper and lower jaws, and surrounding areas (e.g., temporomandibular joints, maxillary sinuses, submandibular regions and neck regions). Patients' orthopantomograms are reviewed by qualified RFDS (Queensland Section) employed dental practitioners who generate reports within their clinical notes. While dental practitioners have excellent diagnostic skills, comprehensive documentation of radiographic findings can be time consuming and difficult to achieve in busy clinical environments, particularly those where the RFDS (Queensland Section) Mobile Dental Unit operates. As a result, the extent of information recorded may vary between dental practitioners and the nature of the clinical presentation. For example, the presence or absence of dental caries may be assessed by the respective dental practitioner as part of routine care but not recorded in their orthopantomogram report, unless associated with some abnormality and/or more significant pathology. This is consistent with what would be expected in most metropolitan fixed‐location dental practices that are also reporting their images in‐house and not via a specialist service. In contrast, oral and maxillofacial radiologists, who have additional specialty training in dental diagnostic imaging and interpretation, focus on completing comprehensive reviews without the same time constraints.

Although orthopantomograms enable clinicians to detect abnormalities and/or disease processes, image quality attributable to positional and/or exposure faults (e.g., “ghost shadows,” magnification variations, etc.) can often impact the diagnostic acceptability of images (Rushton and Horner 1996; Rushton et al. 1999; Schiff et al. 1986; Brezden and Brooks 1987). Prior research has also shown that orthopantomograms interpreted by non‐specialists can produce false‐negative and false‐positive results (e.g., McNab et al. 2015)). In regional and remote clinical services, false‐negative results may provide patients with a false sense of reassurance and can delay other treatment‐seeking whereas false‐positive results can result in significant direct and indirect costs including unnecessary days out of role travelling several hundred kilometres for referrals out‐of‐community. Structured reporting tools have been proposed as a way of improving the consistency and comprehensiveness of diagnostic imaging reports, which may reduce the potential adverse outcomes associated with missed findings and unnecessary referrals.

Similarly, some public and private dental practices use external radiology services to ensure access to specialist advice and comprehensive specialist orthopantomogram reviews. In this context, “referral” denotes a structured pathway that standardises care by providing all practitioners within a given service a consistent point of contact for specialist input. Without such pathways, clinicians rely on their own networks, which vary depending on factors such as years of practice and size of their patient catchment. Unlike fixed‐base services, for the Mobile Dental Unit, this catchment and referral network is equivalent to 1.72 million square kilometres making reliance on informal networks challenging.

Whilst RFDS (Queensland Section)'s capacity to provide orthopantomogram reports is sound clinical practice, the incremental benefits of using a structured orthopantomogram reporting tool compared to current practice; and the potential scope of a formal specialist radiologist referral pathway are yet to be explored. As a result, this study piloted a structured orthopantomogram reporting tool:

- To examine its inter‐rater concordance between two dental practitioners,
- To compare outputs to current reporting practices, and
- To explore the potential scope of a specialist referral pathway in supporting service delivery for regional and remote communities.

## 2 Materials and Methods

### 2.1 Sample

No participants were actively recruited to this study. Instead, participant records were retrospectively extracted from the RFDS (Queensland Section) electronic clinical management system. Each year, approximately 450 adult patients have orthopantomogram(s) taken as part of their clinical assessment. A random sample of 50 adult patients who consulted the RFDS (Queensland Section) Mobile Dental Unit between January 1, 2023 and July 31, 2023, had an orthopantomogram taken as part of their clinical assessment, and gave their informed consent for their data to be used for research purposes, were sampled for inclusion in this study (e.g., a 22% sampling rate). This study was approved by the University of New England Human Research Ethics Committee (HE24‐078).

### 2.2 Assessments and Procedures

#### 2.2.1 Demographics

Participants' sex, age, and whether they reported being Aboriginal and/or Torres Strait Islander are routinely collected at each RFDS (Queensland Section) dental consultation and were used to characterize the sample. The Australian Statistical Geography Standard Remoteness Structure was used to determine whether participants resided in major cities, inner or outer regional areas, or remote or very remote areas (Australian Bureau of Statistics 2021).

#### 2.2.2 Orthopantomograms

The digital orthopantomogram images collected at participants' respective consultation were extracted from the RFDS (Queensland Section) electronic clinical management system.

#### 2.2.3 Structured Orthopantomogram Reporting Tool

For the purposes of this evaluation, a structured online reporting tool was developed based on “gold standard” orthopantomogram reporting practices (e.g., Mallya and Lam 2018), a review of the literature, and specialist radiologist recommendations (see Supporting Information: Materials 1). This tool was used to assess:

- image quality (e.g., presence of positional and/or exposure faults);
- presence, number and location (quadrants 1–4) of supernumerary teeth;
- positional and/or anatomical abnormalities of non‐third molar teeth;
- presence of third molars;
- presence and location (quadrants 1–4) of caries;
- presence of radiolucencies and opacities;
- presence of possible soft tissue calcifications/ossifications in the submandibular/neck regions;
- presence of potentially significant pathology; and
- whether the orthopantomogram should be selected for specialist radiologist review.

The time taken to complete the structured reporting tool was also recorded.

Two dental practitioners with bachelor's degrees in general dentistry, each with 6+ years of clinical experience and regular involvement in orthopantomogram interpretation, independently used this structured tool to assess all 50 images. The practitioners completed their ratings independently and were blind to each other's assessments. The dentists were also blinded to the information recorded in the routine clinical reports during this phase of data extraction. No formal calibration session was conducted prior to rating; however, both practitioners were experienced with orthopantomogram interpretation. After completing independent assessments, the two dental practitioners case conferenced their findings. The results of the structured reporting tool for patients whom additional specialist radiologist expertise was deemed to be indicated were then case conferenced with a dento‐maxillofacial radiologist.

#### 2.2.4 Orthopantomogram Reports Generated as Part of Routine Care

After using the structured reporting tool for primary data collection, 50% of the orthopantomogram reports that were generated as part of routine care were randomly assigned to each dental practitioner. The structured reporting tool was then used to index the information that was recorded in each of these existing reports.

### 2.3 Analyses

All analyses were completed using STATA v18. Descriptive analyses were used to characterise participant demographics and the distribution of orthopantomogram features. Agreement analyses examined (1) inter‐rater concordance between two dental practitioners using the structured orthopantomogram reporting tool; and (2) inter‐method concordance between structured tool outputs and the information recorded during routine care.

#### 2.3.1 Inter‐Rater Agreement

Each structured orthopantomogram reporting tool item included “Yes,” “No,” and “Unsure” response options, reflecting the interpretive uncertainty that can arise in orthopantomogram assessment. The same three‐category structure was used for the item relating to whether specialist radiologist review was recommended. Overall observed agreement (*P*_*O*_) was calculated as the proportion of ratings in which both practitioners selected the same response category. Cohen's *κ* was used to estimate agreement beyond chance. *κ* values were interpreted according to Landis and Koch, with thresholds of 0–0.20, 0.21–0.40, 0.41–0.60, 0.61–0.80, > 0.81 indicative of slight, fair, moderate, substantial and almost perfect agreement (Landis and Koch 1977). Ninety‐five percent confidence intervals for proportions (e.g., observed agreement) were calculated using a Wald binomial approximation, with upper limits truncated at 1.00 where applicable. Ninety‐five percent confidence intervals for *κ* were calculated using a normal approximation (*κ* ± 1.96*SE), with upper limits truncated at the theoretical maximum.

#### 2.3.2 Inter‐Method Agreement

For concordance analyses between the structured reporting tool and routine clinical reports, the two practitioners' structured tool ratings were collapsed into three categories (Yes, No, and Uncertain) to ensure comparability with the categorical structure of the routine clinical reports. If both practitioners selected the same response category, that category was assigned. When responses differed, discordant pairs were classified as Uncertain to preserve a conservative estimate of agreement and to avoid over‐attributing concordance where clinician interpretation differed. This approach is expected to lower observed agreement and *κ* values relative to alternative coding strategies. A 3 × 3 contingency table was constructed for each orthopantomogram feature, and observed agreement, Cohen's *κ* and 95% confidence intervals were calculated using the same procedures as described for the inter‐rater analyses.

## 3 Results

### 3.1 Sample Demographics

Participant demographics are shown in Table 1. In general, the sample was slightly more female and aged, on average, in their mid‐forties (*R* = 19–74 years; M(SD) = 44.50(15.85); Mdn (IQR) = 42 (29–58)). Sixty percent reported being non‐Indigenous, 10% reported being Aboriginal or Torres Strait Islander and 30% preferred not to provide this information. Patients predominantly resided in remote (36%) and very remote (28%) areas with just over a third residing in outer regional areas (36%).

**Table 1:** Sample demographics (*N* = 50).

	Total sample (*N* = 50)	
	*n*	%
Sex		
Female	26	52.0%
Male	24	48.0%
Aboriginal and/or Torres Strait Islander		
Neither	30	60.0%
Aboriginal and/or Torres Strait Islander	5	10.0%
Prefer not to say	15	30.0%
Remoteness Area		
Major cities	—	—
Inner regional	—	—
Outer regional	18	36.0%
Remote	18	36.0%
Very remote	14	28.0%

### 3.2 Concordance Between Two Dental Practitioners Using the Structured Reporting Tool

#### 3.2.1 Tool Completion Time

On average, the structured reporting tool was completed in 10.25 min (SD = 8.62; Mdn (IQR) = 8.38 (5.65–11.18)).

#### 3.2.2 Inter‐Rater Agreement Using the Structured Tool

Overall, there was an observed agreement of 0.94 (95% confidence interval [CI] = 0.93–0.96) between the two dental practitioners when using the structured orthopantomogram tool (*n* = 707/750), indicating that the tool yielded almost perfect agreement after adjusting for chance (*κ* [95%CI] = 0.89 [0.86–0.92]) (see Table 2).

**Table 2:** Inter‐rater agreement of the structured reporting tool (*N* = 50).

	Presence or absence of OPG feature										
	Agreed: Both yes	Agreed: Both no	Disagreed: Yes/no	Disagreed: No/yes	Agreed: Both unsure	Disagreed: Yes/unsure	Disagreed: No/unsure	Observed agreement	(95%CI)	*κ*	(95%CI)
Image assessments											
Position faults	39	9	2	0	0	0	0	0.96	(0.91–1.00)	0.88	(0.71–1.00)
Exposure faults	12	37	0	0	1	0	0	1.00		1.00	(1.00–1.00)
Supernumerary teeth present	0	50	0	0	0	0	0	1.00		*	
Not related to 3rd molars											
Positional abnormalities	1	49	0	0	0	0	0	1.00		1.00	(1.00–1.00)
Anatomical abnormalities	3	46	0	0	0	0	1	0.98	(0.94–1.00)	0.82	(0.48–1.00)
Third molars present	31	19	0	0	0	0	0	1.00		1.00	(1.00–1.00)
Caries present											
Q1	17	23	1	0	9	0	0	0.98	(0.94–1.00)	0.97	(0.91–1.00)
Q2	15	25	0	0	9	0	1	0.98	(0.94–1.00)	0.97	(0.90–1.00)
Q3	16	24	0	0	8	0	2	0.96	(0.91–1.00)	0.93	(0.84–1.00)
Q4	22	19	0	0	7	1	1	0.96	(0.91–1.00)	0.93	(0.85–1.00)
Upper and lower anterior regions											
Radiolucencies present	7	39	1	0	3	0	0	0.98	(0.94–1.00)	0.94	(0.90–1.00)
Opacities present	11	36	1	1	0	0	1	0.94	(0.87–1.00)	0.84	(0.66–1.00)
Submandibular/neck regions											
Possible soft tissue calcifications/ossifications	15	29	4	1	0	0	1	0.88	(0.79–0.97)	0.74	(0.54–0.93)
Possible significant pathology	39	6	3	0	1	1	0	0.92	(0.84–1.00)	0.72	(0.46–0.98)
Specialist review indicated	8	22	16	1	0	1	2	0.60	(0.46–0.74)	0.20	(−0.07 to 0.47)
Total	236	433	28	3	38	3	9	0.94	(0.93–0.96)	0.89	(0.86–0.92)

*Note:* CIs are not reported for proportions equal to 1.00 because the standard error is zero and the CI collapses to a point estimate; *κ could not be estimated for this feature due to perfect agreement and zero variability in ratings (i.e., both raters selected “No” for all cases).

Abbreviations: CI, confidence interval; OPG, orthopantomogram.

With regard to individual orthopantomogram findings, there was almost perfect to perfect agreement on image quality indicators (e.g., positional and/or exposure faults), the presence of supernumerary teeth, non‐third molar‐related positional and anatomical abnormalities, the presence of third molars and caries, and the detection of radiolucencies and opacities in the upper and lower anterior regions (*R*_*κ*_ = 0.82 – 1.0). There was substantial agreement for possible soft tissue calcifications/ossifications in the submandibular and neck regions (*κ* [95% CI] = 0.74 [0.54–0.93]) and for the presence of possible significant pathology (*κ* [95%CI] = 0.72 [0.46–0.98]). In contrast, agreement on whether a specialist review was required was at the lower boundary of the fair range (*κ* [95%CI] = 0.20 [−0.07 to 0.47]). Some *κ* estimates had wide confidence intervals due to small cell counts and the low prevalence of certain findings; these estimates should therefore be interpreted with caution.

##### 3.2.2.1 Sources of Uncertainty and Disagreement

Caries assessment was the largest source of uncertainty, accounting for 76.00% of bivariate comparisons involving “Unsure” ratings (*n* = 38/50). Among the 31 “Yes/No” disagreements, most related to referral recommendations (*n* = 17/31, 54.84%), followed by possible soft tissue calcifications or ossifications (*n* = 5/31, 16.12%).

##### 3.2.2.2 Descriptive Orthopantomogram Findings

###### 3.2.2.2.1 Image Quality

Positional faults were common (*n* = 39/50, 78.00%) as were exposure faults (*n* = 12/50, 24.00%).

###### 3.2.2.2.2 Clinical Features

No participants had supernumerary teeth, and few had non‐third molar‐related positional or anatomical abnormalities (8.00%). In contrast, both dental practitioners agreed that 66.00% (*n* = 33/50) of participants had at least one carious tooth in at least one quadrant and 42.00% (*n* = 21/50) had caries in multiple quadrants (i.e., *n* = 21/50 had at least one caries in ≥ 2 quadrants, 24.00% (*n* = 12/50) had caries in ≥ 3 quadrants and 8.00% (*n* = 4) had caries in all quadrants). Practitioners also agreed that possible soft tissue calcifications/ossifications were present in 30.00% of participants (*n* = 15/50), and that possible significant pathology was present in 78.00% of orthopantomograms (*n* = 39/50).

##### 3.2.2.3 Specialist Radiologist Review of Orthopantomograms

Using the structured online tool, both dental practitioners agreed that 16.00% of images should undergo specialist radiologist review (*n* = 8/50; *P*_*O*_ [95%CI] = 0.60 [0.46–0.74]). They disagreed on whether an additional 17 images should be selected for specialist radiologist review (e.g., 16 “Yes/No” disagreements, and one uncertain image). As a result, 25 images (i.e., 50%) were subsequently reviewed by the specialist radiologist.

Upon review, the specialist radiologist concluded that a referral was indicated in three of these cases (i.e., 12% of the 25 selected for specialist radiologist review and 6% of the orthopantomograms included in this pilot study). Discussion of the 22 instances where referral was not indicated identified opportunities to reduce future out‐of‐community referrals through targeted education, particularly regarding opacities noted incidentally and radiographic anatomy. These proportions reflect the subset of cases that were selected for specialist radiologist review and should not be interpreted as the overall prevalence of cases requiring specialist referral within the service.

### 3.3 Agreement Between the Structured Reporting Tool and Routine Reporting Practices

Overall, there was an observed agreement of 0.68 (95%CI = 0.65–0.72) between the structured tool ratings and routine clinical reports. After adjusting for chance, agreement was fair (*κ* [95%CI] = 0.33 [0.29–0.37], see Tables 3 and 4). Routine reports recorded fewer features overall, which is consistent with the lower agreement for features that were identified more consistently using the structured tool. Most differences likely reflect the structured tool prompting more detailed documentation rather than the quality of routine reporting practices.

**Table 3:** Distribution of structured tool and routine report response combinations for each orthopantomogram feature (*N* = 50).

	Agreed: ST = Yes RR = Yes	Agreed: ST = No RR = No	Disagreed: ST = Yes RR = No	Disagreed: ST = No RR = Yes	Agreed: ST = Uncertain RR = Uncertain	Disagreed: ST = Yes RR = Uncertain	Disagreed: ST = No RR = Uncertain	Disagreed: ST = Uncertain RR = Yes	Disagreed: ST = Uncertain RR = No
Supernumerary teeth present	0	50	0	0	0	0	0	0	0
Not related to third molars									
Positional abnormalities	0	46	1	3	0	0	0	0	0
Anatomical abnormalities	0	43	3	3	0	0	0	1	0
Third molars present	20	18	11	0	0	0	1	0	0
Caries present									
Q1	9	21	7	2	2	1	0	0	7
Q2	5	24	10	1	2	0	0	0	7
Q3	7	23	8	0	2	1	1	0	7
Q4	3	15	18	2	1	1	2	2	5
Upper and lower anterior regions									
Radiolucencies present	2	37	5	1	0	0	1	0	4
Opacities present	0	33	11	1	0	0	2	0	3
Submandibular/neck regions									
Possible soft tissue calcifications/ossifications	0	29	15	0	0	0	0	0	6
Possible significant pathology	29	3	9	3	1	1	0	3	1
Specialist review indicated	1	22	7	0	1	0	0	0	19
Total	76	364	107	16	9	4	7	6	63

Abbreviations: RR, routine reports; ST, structured tool.

**Table 4:** Inter‐method agreement between the structured tool ratings and routine clinical reports (*N* = 50).

	Observed agreement	(95%CI)	*κ*	(95%CI)
Supernumerary teeth present	1	—	—	—
Not related to 3rd molars				
Positional abnormalities	0.92	(0.84–1.00)	0.47	(−1.00 to 0.94)
Anatomical abnormalities	0.86	(0.75–0.97)	0.06	(−0.59 to 0.71)
Third molars present	0.76	(0.63–0.89)	0.55	(0.33–0.77)
Caries present				
Q1	0.65	(0.50–0.80)	0.40	(0.17–0.63)
Q2	0.63	(0.48–0.78)	0.30	(0.05–0.56)
Q3	0.65	(0.50–0.80)	0.38	(0.14–0.62)
Q4	0.39	(0.24–0.54)	0.02	(−0.20 to 0.23)
Upper and lower anterior regions				
Radiolucencies present	0.78	(0.66–0.90)	0.19	(−0.23 to 0.61)
Opacities present	0.66	(0.52–0.80)	−0.07	(−0.49 to 0.34)
Submandibular/neck regions				
Possible soft tissue calcifications/ossifications	0.58	(0.44–0.72)	−0.16	(−0.33 to 0.33)
Possible significant pathology	0.66	(0.52–0.80)	0.19	(−0.13 to 0.50)
Specialist review indicated	0.48	(0.34–0.62)	0.08	(−0.16 to 0.33)
Total	0.69	(0.65–0.72)	0.33	(0.29–0.37)

*Note:* Wide confidence intervals for selected *κ* estimates reflect sparse data and small cell counts and should be interpreted with caution. In instances of very low prevalence or perfect agreement, *κ* may be unstable.

Abbreviation: CI, confidence interval.

## 4 Discussion

Among a random sample of 50 RFDS (Queensland Section) Mobile Dental Unit patients, this study reported on orthopantomograms' quality and characteristics and found that a structured reporting tool yielded almost perfect agreement between two dental practitioners but only fair agreement with the information recorded as part of routine clinical care. Based on the structured tool, the dental practitioners agreed that 50% of sampled images required or may have benefited from specialist review. Upon specialist review, referral was indicated in 6% of sampled cases (*n* = 3). These findings have implications for radiographic reporting practices and for the provision of oral health services in regional and remote communities.

### 4.1 Orthopantomogram Quality and Characteristics

Consistent with the broader literature, these data affirm that orthopantomogram quality can be compromised in routine practice, (Rushton and Horner 1996; Rushton et al. 1999; Schiff et al. 1986; Brezden and Brooks 1987) with both dental practitioners agreeing that positional (78%) and exposure faults (24%) were common. Ideally, clinicians would take and interpret technically perfect images. However, multiple factors including patients' anatomical build, their mobility, and clinician training can make this difficult. Hence, provided that the image is diagnostically acceptable, the image can still be useful. Indeed, it would be unethical and against the “as low as reasonably achievable” principle to take multiple exposures (Australian Dental Association 2019) for the purposes of achieving a technically perfect image if the information sought by the clinician was evident. Nonetheless, results suggest that further training on patient positioning could increase image quality and reduce the need for multiple exposures.

It is also important to consider these findings within the broader public health context. Australians living in regional and remote areas experience greater burden of disease attributable to caries and periodontal causes than those in major cities (Australian Institute of Health and Welfare 2023a; Australian Government Department of Health 2012; Australian Institute of Health and Welfare 2007; Crocombe et al. 2013). Similarly, this study found relatively high rates of caries, periodontal disease and apical inflammatory radiolucencies, confirming the need for this regional and remote clinical service. Yet, results indicate that relying on orthopantomograms to assess caries introduces a non‐trivial source of uncertainty. Proximal surface caries are often more difficult to assess using orthopantomograms due to the horizontal overlap of contacts, poorer resolution and/or distortion of the image in these regions, with bitewing radiographs being the recommended imaging option (Australian Dental Association 2019). It is however, important to note that the disagreements and uncertainty with respect to caries are less practically consequential than uncertainty relating to other abnormalities, as visual examination of the dentition and/or intraoral radiographs can readily resolve these issues without specialist referral.

### 4.2 Inter‐Rater Agreement of a Structured Orthopantomogram Reporting Tool

Overall, the structured reporting tool produced almost perfect inter‐rater agreement (*κ* [95%CI] = 0.89 [0.86–0.92]). Maximising the inter‐rater consistency and reliability of orthopantomogram reports is particularly important for the communities served by the Mobile Dental Unit. Patients in regional and remote areas tend to have limited local access to dental services, (Australian Institute of Health and Welfare 2023b) and can incur significant costs when receiving care out‐of‐community. These treatment‐seeking barriers reduce the likelihood of regular check‐ups and preventative care (Crocombe et al. 2013; Curtis et al. 2007). This means that care is often sought when dental disease is painfully acute and urgent, rather than preventable and manageable by less invasive and costly procedures. The RFDS (Queensland) Mobile Dental Unit aims to reduce these barriers and improve patient outcomes by providing local, free and annual services.

Variability in orthopantomogram interpretations has been described in the literature, (McNab et al. 2015) and although this pilot was not designed to evaluate diagnostic accuracy, these concepts are clinically relevant for regional and remote service delivery. In major cities, the consequences of a missed finding may be mitigated by patients' proximity to care and opportunities for follow‐up. In contrast, regional and remote barriers to care mean that delays can be prolonged, and subsequent appointments may not occur for many months. Conversely, identifying an abnormality or acute pathology when none is present can lead to unnecessary out‐of‐community referrals for diagnostic imaging (e.g., computed tomography [CT] scans) and specialist appointments. Patients living in Queensland's northwest, for example, may face a four (or more) hour return drive to Mt Isa for imaging services and/or 24 (or more) hour return drive to the nearest dental specialist in Townsville, with associated time, fuel and accommodation costs. Tropical weather trends further complicate access, as roads may be inaccessible during the wet season. Implementing a structured reporting tool that demonstrates high agreement between clinicians could help minimise inconsistencies in reporting and provide a clearer basis for determining when specialist review is, and is not, indicated. While further validation is needed before implementation decisions are made, structured tools offer a way to support more consistent reporting and may, if feasible within service workflows, contribute to improved outcomes for regional and remote patients.

### 4.3 Comparison of the Structured Tool and Routine Reporting Practices

These data provide proof of concept that structured tools can strengthen reporting consistency and increase the level of detail documented in patients' orthopantomogram reports thereby supporting the identification of potentially significant pathologies. Importantly, the lower agreement observed between the structured tool and routine clinical reports should not be interpreted as inferiority of routine practice, but rather as reflecting differences in reporting structure, granularity, and the real‐time clinical context in which routine reports are generated. Within this context, implementing the structured reporting tool in routine practice could provide a standardised reporting framework for Mobile Dental Unit practitioners and prompt more comprehensive documentation including anatomical structures and findings outside of the dentition. However, any implementation needs to be considered within the broader operational environment of a mobile regional and remote service. A standard RFDS (Queensland Section) Mobile Dental Unit examination appointment is scheduled for 30 min, and the structured tool required approximately 10 min per report. In addition to appointment‐level time pressures, the service operates with a small travelling workforce, long transit distances, and fixed community schedules. Integrating additional reporting tasks into this workflow therefore has direct implications for daily throughput and overall service reach. Consequently, implementing the structured reporting tool within current workforce capacity is unlikely to be feasible without reducing daily consultations, increasing days spent in each community or modifying the tool to reduce completion time. While completion time is likely to decrease with familiarity, a streamlined or digital version of the tool with dropdown options, automated prompts or decision‐support features may further shorten the reporting process. Further research is needed to assess these options alongside clinician acceptability prior to implementing any changes in routine practice.

### 4.4 Specialist Review of Orthopantomograms

Using the structured orthopantomogram reporting tool, two dental practitioners agreed that a specialist radiologist review was required for 16% of sampled patients and potentially indicated for a further 34%. Of the 25 images reviewed by the specialist radiologist, all but three referrals were not indicated. Of these three images, two were considered “reasonable to refer” and one required referral. In comparison, one referral occurred in routine care. Many of the cases selected for specialist review that did not ultimately warrant referral related to uncertainty regarding the pterygomaxillary fissure. Implementing a referral advice pathway could reduce the likelihood of unnecessary referrals by providing practitioners the opportunity to discuss any concerns and/or uncertainty about orthopantomogram findings with an identified specialist prior to referring the patient for out‐of‐community services. This pathway could also enable dental practitioners to receive informal education, which may support their knowledge and confidence reporting on orthopantomograms. This knowledge base would necessarily be applied to the orthopantomogram reports of future patients, thereby further reducing the need for specialist advice and increasing reporting consistency and supporting clinical decision‐making.

### 4.5 Strengths and Limitations

This study has several key strengths. By focusing on patients seeking dental care in outer regional and remote areas of Australia, a patient group known to experience a greater burden of dental‐related disease and have less access to clinical services than those living in major cities but for whom there is limited peer‐reviewed data available, our findings offer novel insights for the dental, and the regional and remote literatures. Rigorous methods were also implemented and found that a structured orthopantomogram reporting tool increased the extent to which clinical characteristics were recorded. This is one of the only concordance studies available in this space and certainly one of the only published in the past decade. These data provide preliminary evidence of the potential benefits and limitations of using a structured reporting tool to assess patients' orthopantomograms.

These strengths, however, need to be considered within the context of the following caveats. Although the sample size was sufficient for current purposes, this was a small pilot conducted within a single regional and remote dentist service, and findings may not generalise to other service models or populations. Only participants who received an orthopantomogram were included, which may introduce selection bias if these patients differ systematically from those who did not require radiographic imaging. Additionally, only those images selected by one or both dentists were reviewed by the specialist, meaning that specialist verification was partial rather than comprehensive. Routine clinical reports also served as an imperfect comparator. Their purpose is to support point‐of‐care decision‐making rather than to provide a structured record of every anatomical feature. Accordingly, the “fair” agreement observed between structured and routine reports is likely to reflect the structured tool prompting more detailed documentation rather than shortcomings in the quality of existing reporting practices. No formal calibration session occurred prior to data extraction, and although both dental practitioners had extensive clinical experience, the absence of pre‐rating alignment may have contributed to variability in item‐level agreement. Additionally, both practitioners were experienced in orthopantomogram interpretation, and agreement may differ in less experienced practitioners, which may limit generalisability to broader workforce settings. Replication of reporting trends is needed prior to any implementation efforts, as is further investigation of the perceived benefits and barriers to implementing such a reporting tool among a broader sample of dental practitioners.

## 5 Conclusions

These data provide insights into the role that the RFDS (Queensland Section) has in providing essential acute, preventative and restorative dental services to otherwise underserved regional and remote communities. Structured tools encourage comprehensive orthopantomogram reports and enhance inter‐rater concordance, but their implementation needs to be practical within the constraints of service delivery. The findings indicate that structured reporting can support consistency in documenting clinically relevant features, although time and workflow considerations are likely to influence feasibility. A specialist advice pathway could reduce unnecessary referrals to out‐of‐community services, and support dentists' confidence when completing orthopantomogram reports.

## Author Contributions

**Stevie Dilley:** conceptualization, investigation, resources, writing – review and editing, funding acquisition, project administration. **Lynlee Tatnell:** investigation, writing – review and editing. **Paul Monsour:** methodology, investigation, writing – review and editing. **Jacob O'Gorman:** conceptualization, methodology, resources, writing – review and editing, funding acquisition. **Megan J. Hobbs:** conceptualization, methodology, analysis, resources, data curation, writing – original draft, writing – review and editing, supervision, funding acquisition, project administration.

## Ethics Statement

This research was approved by the University of New England's Human Research Ethics Committee (HE24‐078).

## Consent

No participants were actively recruited for this evaluation. Instead, participants data were sampled from clinical records. All sampled participants provided their informed consent for their data to be used for quality assurance and/or research purposes. This occurred as part of RFDS (Queensland Section) dental service's standard intake procedures that occur independent of this evaluation.

## Conflicts of Interest

The authors declare no conflicts of interest.

## Supporting information

Supporting File

## Data Availability

No study data will be shared with any third parties. No study data will be shared with any third parties as participants' consent was limited to the use of their data by Royal Flying Doctor Service (Queensland Section) employees for research or quality assurance purposes.
