# Supplementary material for: Concordance of a Structured Orthopantomogram Reporting Tool Between Dental Practitioners, and Compared to Routine Reports Within a Regional and Remote Service
Source: Clin Exp Dent Res. 2026 Jul 16;12(4):e70366. doi: 10.1002/cre2.70366 (PMC13375152; doi:10.1002/cre2.70366)
Supplement: Supplementary file 1 — Supporting File [file CRE2-12-e70366-s001.docx]

Supplementary Materials: Structured Orthopantomogram Reporting Tool Used for Image Assessment and Referral Decisions

Dilley et al. Concordance of a structured orthopantomogram reporting tool between dental practitioners, and compared to routine reports within a regional and remote service. *Clinical and Experimental Dental Research.*

Supplementary Table S1a. Administrative and Image Orientation Details

| Item Number | Reporting Tool Item | Categorical Response Option(s) | Free Text Field Option(s) | Skip Logic |
| --- | --- | --- | --- | --- |
| Q1 | Participant ID | N/A | Free text data entry | N/A |
| Q2 | Which researcher is responsible for data entry? | Initials of the research team | N/A | N/A |
| Q3 | Is the OPG orientated correctly? *i.e., the L marker is on the right hand side of the OPG as you look at the film* | Yes/No | N/A | N/A |

Supplementary Table S1b. Image Quality Assessment

*Before you begin your report, mask out all extraneous light around the radiograph, so that only the radiograph is illuminated on the monitor. Ideally, reduce the ambient lighting in the room. You are now ready to begin.*

| Item Number | Reporting Tool Item | Categorical Response Option(s) | Free Text Field Option(s) | Skip Logic |
| --- | --- | --- | --- | --- |
| Q4 | Does the film have positional faults | Yes/No/Unsure | Please specify … | N/A |
| Q5 | Does the film have exposure faults | Yes/No/Unsure | Please specify … | N/A |
| Q6 | What is the quality of the image? | - Excellent (i.e., free of positioning and exposure errors) - Diagnostically acceptable (i.e., had errors that did not detract from the OPG’s diagnostic utility) - Unacceptable (i.e., had errors that did detract from the OPG’s diagnostic utility) | N/A | If Q6 = Unacceptable 🡪 Q7;  If Q6 = Excellent or acceptable 🡪Q8 |
| Q7 | Please specify the areas of the film that make the OPG diagnostically unacceptable | N/A | Please specify … | N/A |

Supplementary Table S1c. Dentition Count and Non-Third Molar Abnormalities

| Item Number | Reporting Tool Item | Categorical Response Option(s) | Free Text Field Option(s) | Skip Logic |
| --- | --- | --- | --- | --- |
| Q8 | Count the patient's teeth. Does the patient have any supernumerary teeth? | Yes/No/Unsure | Unsure 🡪 Please specify … | If Q8 = Yes 🡪Q9;  If Q8 = No or Unsure 🡪 Q11 |
| Q9 | How many supernumerary teeth does the patient have? | N/A | Please specify … | N/A |
| Q10 | Where are the supernumerary teeth located? | N/A | Please specify … | N/A |
| Q11 | In relation to all of the patient's teeth (excluding third molars), are there any positional abnormalities? | Yes/No/Unsure | Unsure 🡪 Please specify … | If Q11 = Yes 🡪 Q12;  If Q11 = No/Unsure 🡪 Q13 |
| Q12 | What positional abnormalities are present? | N/A | Please specify … | N/A |
| Q13 | Are there any anatomical abnormalities present (excluding third molars)? | Yes/No/Unsure | Unsure 🡪 Please specify … | If Q13 = Yes 🡪 Q14;  If Q13 = No/Unsure 🡪 Q15 |
| Q14 | What anatomical abnormalities are present? | N/A | Please specify … | N/A |

Supplementary Table S1d. Systematic Review of Radiograph Features and Referral Decision

*Now, systematically review all areas on the radiograph.*

| Item Number | Reporting Tool Item | Categorical Response Option(s) | Free Text Field Option(s) | Skip Logic |
| --- | --- | --- | --- | --- |
| Q15 | Are there third molars present? | Yes/No/Unsure | Unsure 🡪 Please specify … | If Q15 = Yes 🡪Q16;  If Q15 = No or Unsure 🡪 Q17 |
| Q16 | Are the third molars present in the ...?   - Quadrant 1 - Quadrant 2 - Quadrant 3 - Quadrant 4 | Yes/No | N/A |  |
| Q17 | Are there signs of caries radiographically in the ... ?   - Quadrant 1 - Quadrant 2 - Quadrant 3 - Quadrant 4 | Yes/No/Unsure | N/A | N/A |
| Q18 | In relation to the upper and lower anterior regions, are there radiolucencies? | Yes/No/Unsure | Unsure 🡪 Please specify … | If Q18 = Yes 🡪 Q19; If Q18 = No/Unsure 🡪 Q22 |
| Q19 | Are the radiolucencies projectional? | Yes/No/Unsure | Yes/Unsure 🡪 Please specify … | N/A |
| Q20 | Are the radiolucencies anatomical? | Yes/No/Unsure | Yes/Unsure 🡪 Please specify … | N/A |
| Q21 | Are the radiolucencies pathological? | Yes/No/Unsure | Yes/Unsure 🡪 Please specify … | N/A |
| Q22 | In relation to the upper and lower anterior regions, are there opacities? | Yes/No/Unsure | Unsure 🡪 Please specify … | If Q22 = Yes 🡪 Q23; If Q22 = No/Unsure 🡪 Q26 |
| Q23 | Are the opacities projectional? | Yes/No/Unsure | Yes/Unsure 🡪 Please specify … | N/A |
| Q24 | Are the opacities anatomical? | Yes/No/Unsure | Yes/Unsure 🡪 Please specify … | N/A |
| Q25 | Are the opacities pathological? | Yes/No/Unsure | Yes/Unsure 🡪 Please specify … | N/A |
| Q26 | Are there possible soft tissue calcifications/ossifications in the submandibular/neck regions? | Yes/No/Unsure | Unsure 🡪 Please specify … | N/A |
| Q27 | Please specify the location of the possible soft tissue calcifications/ossifications in the submandibular/neck regions? | - Tonsillar - Submandibular - Lymph nodes - Carotid - Ossified stylohyoid ligament - Other - Unsure | All responses 🡪 Please specify … | N/A |
| Q28 | Considering your overall interpretation of the OPG, does the patient have any potential significant pathology? | Yes/No/Unsure | Yes/Unsure 🡪 Please specify … | N/A |
| Q29 | Based on your review of the OPG, should the patient’s OPG be referred to a specialist radiologist? | Yes/No/Unsure | All responses 🡪 Please specify … | N/A |
